# Comparison of Polysaccharides as Coatings for Quercetin-Loaded Liposomes (QLL) and Their Effect as Antioxidants on Radical Scavenging Activity

**DOI:** 10.3390/polym12122793

**Published:** 2020-11-26

**Authors:** Manuel Román-Aguirre, César Leyva-Porras, Pedro Cruz-Alcantar, Alfredo Aguilar-Elguézabal, María Zenaida Saavedra-Leos

**Affiliations:** 1 Doctorado Institucional en Ingeniería y Ciencia de los Materiales, Universidad Autónoma de San Luis Potosí, San Luis Potosí 78210, Mexico; iq_manuelroman@yahoo.com; 2 Laboratorio Nacional de Nanotecnología (NanoTech), Centro de Investigación en Materiales Avanzados S.C. (CIMAV), Chihuahua 31136, Mexico; cesar.leyva@cimav.edu.mx; 3 Coordinación Académica Región Altiplano (COARA), Universidad Autónoma de San Luis Potosí, Matehuala 78700, Mexico; pedro.cruz@uaslp.mx; 4 Departamento de Ingeniería y Química de Materiales, Centro de Investigación en Materiales Avanzados, S.C. (CIMAV), Chihuahua 31136, Mexico; alfredo.aguilar@cimav.edu.mx

**Keywords:** loaded liposomes, antioxidant properties, polysaccharides coating, inulin, lactose, quercetin

## Abstract

Liposomes are microstructures containing lipid and aqueous phases employed in the encapsulation and delivery of bioactive agents. Quercetin-loaded liposomes (QLLs) were coated with three different polysaccharides and then tested as radical scavengers. Lactose (LCQLL), chitosan (CCQLL), and inulin (ICQLL) were employed as coating materials. Particle size determined by light scattering, showed primary size of 200 nm for all samples, while a secondary particle size of 600 nm was observed for CCQLL. Scanning electron microscopy (SEM) evidenced particle aggregation with the addition of the polysaccharide coating. Transmission electron microscopy (TEM) revealed the layered microstructure of liposomes composed of at least two layers, and primary particle size below 100 nm. QLL showed higher antioxidant activity than the coated liposomes. This behavior was attributed to the chemical interaction between quercetin and the corresponding coating polysaccharide in the layered structure, which traps the quercetin and keeps it unavailable for radical scavenging. From the three polysaccharides, lactose showed a better performance as coating material in the antioxidant activity, which suggested that the smaller size of the disaccharide molecule resulted in a faster releasing of the quercetin in the solution. Thus, LCQLL is an advantageous way to deliver quercetin for antioxidant purposes, where the low stability in delivered media of quercetin loaded liposomes is commonly compromised.

## 1. Introduction

One of the biggest challenges facing in food industry is the rapid deterioration of foods, caused mainly by the growth of microorganisms, lipid oxidation, or enzymatic self-decomposition [1]. Oxidation causes the loss of nutritional value, color, and texture in foods [2,3]. In meat products, lipid oxidation leads to the formation of hydroperoxides and volatile compounds like aldehydes and ketones, provoking a characteristic rancidity smell [4,5,6]. In fruits and vegetables, the deterioration caused by enzymatic oxidation or oxidative stress, is extremely fast, being observed as darkening, loss of texture, and alteration of taste, few hours after harvest or even faster once the peel is removed [7]. In order to counteract this effect, the use of synthetic antioxidants such as butylated hydroxytoluene (BHT) has been implemented, although its consumption has been linked to toxicological effects [8,9]. Consequently, the current trend is the replacement of synthetic antioxidants with naturally occurring ones. Natural antioxidants can be found in some essential oils of herbs and spices such as oregano, cinnamon, rosemary, marjoram or clove, all of them containing compounds such as terpenes (carvacrol, citral or thymol), or aldehydes (cinnamaldehyde) [10,11]. Another natural source rich in antioxidants are red fruits i.e., berries, grapes, and pomegranates, which are abundant in beneficial compounds such as terpenoids (resveratrol), and phenolics (anthocyanins, phenolic acids, flavan-3-oles and flavonoids) [12]. Among the dietary flavonoids, quercetin (2-(3,4-dihydroxyphenyl)-3,5,7-trihydroxy-4*H*-chromen-4-one) is one of the most abundant [13,14]. However, because of the easy decomposition of quercetin upon exposure to environmental elements such as oxygen, temperature and ultraviolet light [15,16,17], it is necessary to encapsulate it with protective vehicles or transport agents that may form a protective film or coating [18]. This problem has also been studied for quercetin applications as anti-cancer agent, being its low oral availability and fast gastrointestinal digestion the main restrains for its use as direct oral drug [19].

Liposomes are self-assembled spherical aggregates, composed of a phospholipid molecule shell (typically one or more bilayers of lecithin) enclosing an aqueous phase core containing a bioactive compound. Since liposomes are prepared in liquid media, the experimental variables affecting the stability may include the pH, temperature of solution and melting point of the phospholipid, among others [20]. Recently, encapsulation in the form of liposomes has emerged as a potential solution to the deterioration and preservation of bioactive compounds [15,21,22]. Due to the presence of both lipid and aqueous phases, these microstructures represent an emerging technology for the encapsulation and delivery of bioactive agents, either hydrophilic or hydrophobic materials, including antioxidants, antimicrobials and nutraceuticals [23,24,25,26]. Thus, in order to increase the stability and improve the release of the active load, liposomes may be coated with other biocompatible compounds. Based on the biocompatibility, low or non-toxicity, and neutral organoleptic properties, several materials have been employed as coatings for liposomes, including glycols, chitosan, and polysaccharides [23,27,28].

Chitosan, poly(D-glucosamine), is a biopolymer with excellent biocompatibility, obtained from deacetylated chitin, and found in the shell of crustaceans like shrimps and lobsters. The positively charged nitrogen of chitosan in acidic solution, prompts this biopolymer to undergo electrostatic attraction to negative charges, like the phosphate group in lecithin. Because of this, chitosan-coated liposomes have been extensively studied [23,24,29,30]. For example, chitosan-coated liposomes loaded with bioactive molecules such as quercetin or carotenoids have shown good stability and release rates [23]. Pectin and polygalacturonic acid do not have a positive charge like chitosan, yet nevertheless they have shown good stability, encapsulation efficiency, and release rate when studied as coating materials for loaded liposomes [25]. Despite the good characteristics of chitosan, the preparation of liposomes coated with this polymer has several limitations, such as the relatively high viscosity of solutions, the low pH required for its solubility and difficulty to eliminate excess of chitosan from the liposomal surface. Alternatively, the use of polysaccharides soluble at neutral pH and with low viscosity can be alternatives to replace the use of chitosan. Polysaccharides’ interaction with the liposome wall affects the release rate of bioactive molecules. In turn, this is dependent on crystallinity, molecular weight, stability and intermolecular attractions of the polysaccharide [22,24,28,30,31,32,33]. Lactose is a disaccharide formed by galactose and glucose subunits. Lactose molecules associated with ceramide (lactosylceramide) have been used as liposome coatings mainly in transporting bioactive molecules inside specific cells to prevent them from being identified as foreign bodies and being destroyed by the mechanisms of the immune system [34,35]. Inulin is a polysaccharide with probiotic properties, consisting of fructose units terminated by end glucose groups. Inulin has been mainly used for encapsulation of antioxidants and other bioactive molecules by the spray drying process [36]. Recently, inulin esterified with stearic acid was employed as a coating of liposomes by penetration of a hydrophobic link of stearic acid inside the phospholipid bilayer [37]. Although in several works, inulin has been included in the hydrophilic phase of the lamellar structure of the liposomes, it is has not been employed as a coating, but as a material that is transported within the liposome. In these works, inulin containing ^3^H has been used as a molecular marker to determine the final location of liposomes in vivo and in vitro tests [38,39]. 

To the best of our knowledge, the antioxidant properties of quercetin-loaded liposomes coated with polysaccharides have not been reported so far. Three coatings were individually studied and compared, neutral low molecular weight-lactose, neutral high molecular weight-inulin, and positively-charged high molecular weight-chitosan. This study is a first investigation towards the improvement of the antioxidant properties and set the basis for future work that may explain the release process of quercetin from the liposome. To the best of our knowledge, the antioxidant properties of quercetin-loaded liposomes coated with inulin or lactose have not been reported so far, and thus, the comparison of polysaccharide coated quercetin loaded liposomes may lead to new knowledge in this field. Therefore, in the present work, chitosan, lactose and inulin were separately employed for coating quercetin-loaded liposomes (QLL). The particle size, morphology and microstructure were characterized, the antioxidant property of the prepared QLL was tested for radical scavenging activity, and the effect of the different coatings compared.

## 2. Materials and Methods

### 2.1. Materials

Soybean phospholipid was purified from food grade lecithin. Methanol, ethanol, acetone, chloroform, acetic acid and sodium phosphate were ACS grade purchased from J.T. Baker (Radnor, PA, USA). Chitosan (80% of deacetylated chitin, and viscosity-average molecular weight of 2.5 × 10^5^ Da), 2,2-diphenyl-1-picrylhydrazyl (DPPH, purity 99.5%) and quercetin (purity 95%) were purchased from Sigma-Aldrich (St. Louis, MO, USA). 

### 2.2. Quercetin-Loaded Liposomes (QLL) Preparation

Prior to liposome preparation, the phospholipid was purified by adapting the method reported by Hasan et al. [28]. Typically, 10 g of food grade commercial lecithin were dissolved in 20 mL of chloroform and poured into 100 mL of acetone. The mixture was cooled to −14 °C and the precipitate decanted by centrifugation. The supernatant liquid was discarded and the solid was washed twice with acetone. Finally, the phospholipid was dried at 25 °C under vacuum for 8 h to eliminate all traces of solvent. The buffer solution was prepared by dissolving 0.01 moles of monobasic sodium phosphate in 0.9 L of deionized water. The pH was adjusted to 7.4 by dropwise addition of 0.1 M NaOH solution. Distilled water was added to make up 1 L of phosphate buffer solution. 

Liposomes were coated with polysaccharides according to the method reported by Hao et al. [23]. The liposome suspension (loaded or unloaded) was mixed with the corresponding polysaccharide solution, i.e., chitosan, lactose or inulin, and separated from the aqueous medium by centrifugation. 

The QLL were prepared as follows. Usually, 60 mg of phospholipid and 2 mg of quercetin were dissolved in 15 mL of solution of methanol and chloroform (1:2). The content of quercetin in the liposomes was below the recommended daily intake dose for humans. The solution was evaporated at 30 °C in a rotary evaporator under reduced pressure to obtain a solvent-free film. The flask containing the film was immersed in an ice bath. The self-assembly of vesicles was done by hydrating the film with 15 mL of phosphate buffer solution under ultrasonic irradiation (3:1 s on/off pulses) during 2 min. Control liposomes (CL), were prepared in a similar way but without adding quercetin to the phospholipid-methanol-chloroform solution. The suspensions obtained were stored at 4 °C and kept in darkness until utilization. 

### 2.3. Chitosan Coated Liposomes

While maintaining magnetic stirring, 2 mL of QLL suspension were added dropwise into 5 mL of chitosan solution (consisting of 0.5 mL of glacial acetic acid and 0.1 g of chitosan in 100 mL of deionized water). Then, the as-obtained coated material was centrifuged at 4 °C and 16,000 RPM during 30 min. The discarded liquid was substituted by a solution of acetic acid (0.5 mL of glacial acetic acid in 100 mL of deionized water) in order to eliminate the excess of chitosan. The washing cycle was repeated twice and the settled solids were dried by lyophilization. Chitosan-coated liposomes were labeled as CCCL, while CCQLL when loaded with quercetin.

### 2.4. Polysaccharide Coated Liposomes

In order to coat QLL with lactose or inulin, the same procedure applied for chitosan coating was followed, except that the acetic acid solution was replaced by deionized water. For example, 2 mL of QLL suspension consisting of 0.1 g of lactose or inulin dissolved in 100 mL of deionized water were added dropwise into 5 mL of polysaccharide solution under magnetic stirring. Coated liposomes were separated by centrifugation at 4 °C and 16,000 RPM during 30 min, then washed twice with deionized water. Solids were dried by lyophilization. Inulin-coated liposomes were identified as ICQLL and ICCL, while lactose-coated liposomes samples were identified as LCQLL and LCCL.

All these coated liposomes were stored at ambient temperature and maintained in darkness until use. Table 1 describes the identification of the samples prepared.

### 2.5. Liposomes Characterization 

#### 2.5.1. Particle Size Distribution

Prior to the centrifugation and washing, part of the liposome suspension was used to measure the particle size distribution (PSD) of the coated liposomes. PSD of samples was determined by light scattering using a Masterziser 2000 particle size analyzer Malvern Instruments (Malvern Panalytical, Malvern, UK) equipped with a 632 nm wavelength laser source. Typically, 12 mL of suspension or coated liposomes suspension were directly poured into the sample port of the instrument. The measurement was repeated three times for each sample. 

#### 2.5.2. Morphology and Microstructure

Morphology and microstructure of liposomes were characterized by scanning electron microscopy (SEM), and transmission electron microscopy (TEM), respectively. 

The SEM analysis consisted in depositing a drop of liposomes solution on a substrate glass. The liquid was dried at room temperature in vacuum conditions by 12 h. In order to reduce charging effects from electron irradiation, dried samples were coated with a thin layer of gold deposited by sputtering. Coated samples were analyzed in a SU3500 SEM (Hitachi, Hitachi city, Japan) operated at 15 kV, and spot size of 40 and low vacuum conditions of 60 Pa. Images were acquired with a secondary electron detector (SEI).

For the TEM analysis, liposome suspensions were first diluted in order to decrease the particle concentration. Commonly, 1 mL of uncoated liposomes suspension or 2 mL of coated liposomes suspension were diluted in 24 mL of deionized water. Then liposomes were stained by mixing 1 mL of the diluted suspension with 1 mL of 2% (wt%) aqueous solution of ammonium molybdate employed as a negative staining agent. Three minutes after mixing, a drop of stained liposome suspension was deposited on a carbon membrane copper grid and dried at room temperature under vacuum conditions. Deposited samples were analyzed on a HT7700 transmission electron microscope (Hitachi), operated at 100 kV.

### 2.6. Radical Scavenging Activity

DPPH radical scavenging activity was evaluated by mixing 1.7 mL of alcoholic DPPH solution (0.1 mmol of DPPH/L in ethanol) with 1.7 mL of lyophilized liposomes suspension. The concentration of liposomes in suspensions varied as 5, 10 and 30 µg/mL. The mixture was left to stand in darkness for 30 min. Then, absorbance was measured at 537 nm using an UV-Vis Evolution 220 spectrometer (Thermo Scientific, Waltham, MA. USA). The scavenging percentage was calculated according to Equation (1):

(1) $$\%\;DPPH*\;=100\times \frac{A_{0}-A_{30}}{A_{0}}$$

where, *A*_0_ is the absorbance of the blank solution i.e., mixture of DPPH and ethanol without liposomes, and *A*_30_ is the absorbance of the of DPPH and ethanol solution containing the liposomes after 30 min. Scavenging activity was determined by triplicate for each sample.

### 2.7. Statistical Analysis

All experiments were performed in triplicate, reporting mean values and standard deviations. One-way analysis of variance (ANOVA) was performed to establish a significance level of 0.05, and the Tukey’s honestly significant difference (HSD) post hoc test was used to determine the difference between means. The statistical analyses were conducted using the IBM SPSS Statistics version 21.0 software (SPSS Inc., Chicago, IL, USA).

## 3. Results and Discussion

### 3.1. Particle Size Distribution

**Figure 1:**
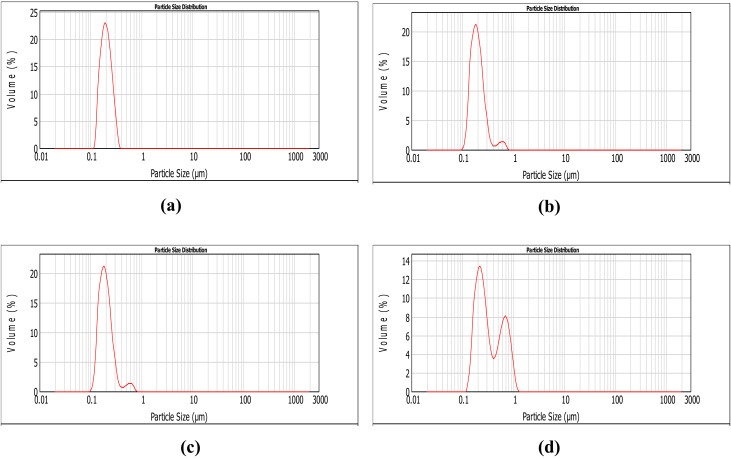
Particle size distribution by light scattering of coated liposomes. (**a**) QLL, (**b**) ICQLL, (**c**) LCQLL and (**d**) CCQLL.

Figure 1 shows the PSD measured by light scattering determined for uncoated and coated quercetin-loaded liposomes. In these figures, the particle size is plotted on the x-axis, while the volume fraction (%) occupied by these particles in the analyzed sample is shown on the y-axis. The average particle size and volume fraction of uncoated (QLL) and coated (ICQLL, and LCQLL) liposomes were similar with values of 185–200 nm, and 21–23%, respectively. 

However, the coated liposomes showed a secondary volume fraction of about 2% with a size distribution on the order of 600–650 nm. Chitosan-coated liposomes (CCQLL) showed slight differences such as a larger primary particle size of 630 nm, lower volume fraction of 13%, and secondary particle size distribution of 690 nm and 8%, respectively. From these results it is evident that inulin and lactose did not affect the primary particle size of the liposomes, while chitosan increased the size. Likewise, the addition of coating materials such as inulin and lactose resulted in the development of a secondary particle size distribution with larger size. Similar results were reported in literature, for example, Hao et al. obtained uncoated liposomes with size of 200–300 nm, while for chitosan-coated liposomes the diameter increased to 700 nm [23]. Park and collaborators prepared chitosan-coated liposomes loaded with resveratrol and observed that the size of uncoated liposomes increased from 200 to 600 nm when using a 0.5% solution of chitosan as coating medium [40]. They explained that several self-assembled layers of chitosan formed on the surface of liposomes increased the diameter of the vesicle. Vural et al. prepared chitosan-coated liposomes loaded with furosemide, and found that particle size increased from 50 to 350 nm when the chitosan concentration was varied from 0.1 to 1% [41]. 

### 3.2. Particle Morphology

**Figure 2:**
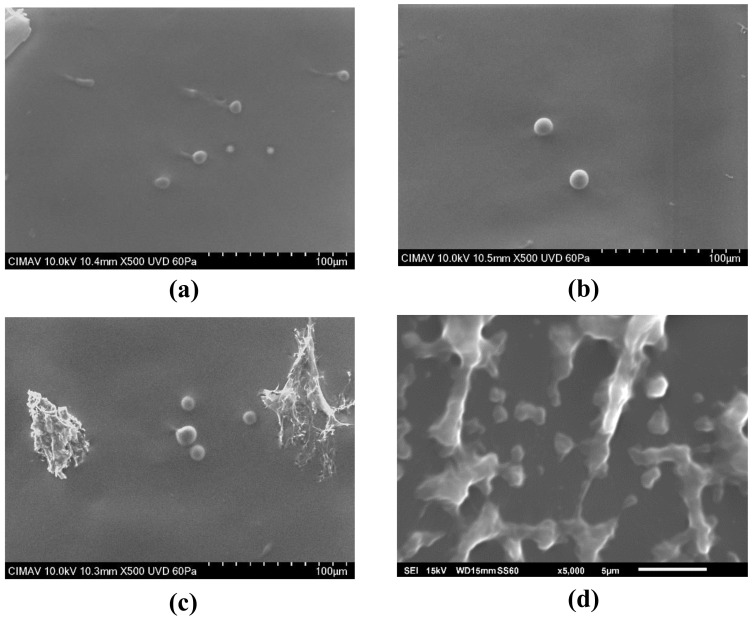
Representative SEM micrographs of CCQLL showing agglomeration of liposomes. (**a**) CL, (**b**) QLL, (**c**) CCCL, and (**d**) CCQLL.

Figure 2 shows representative SEM micrographs of uncoated and coated quercetin-loaded liposomes. Overall, spherically shaped particles with sizes in the range of 1–10 μm were observed, surrounded by a continuous material of irregular morphology. This suggested that the spherical particles corresponded to the agglomerated liposomes, while the continuous material corresponded to the excess lecithin that was not eliminated during the purification process. Several authors have attributed the increase in particle size to the particle agglomeration caused by the addition of the coating material. Li et al. observed that particle aggregation of uncoated samples showed a narrow polydispersity with an agglomerate size of about 4 µm, while for chitosan-coated samples the particle size was about 200 nm with wide polydispersity [42]. In contrast, Romero-Pérez et al. attributed the high polydispersity index to the diverse particle diameter [43]. Barea et al. prepared chitosan-coated liposomes and explained that partial coating of liposomes might cause particle agglomeration due to the interactions between opposite charges [44].

### 3.3. Particle Microstructure

**Figure 3:**
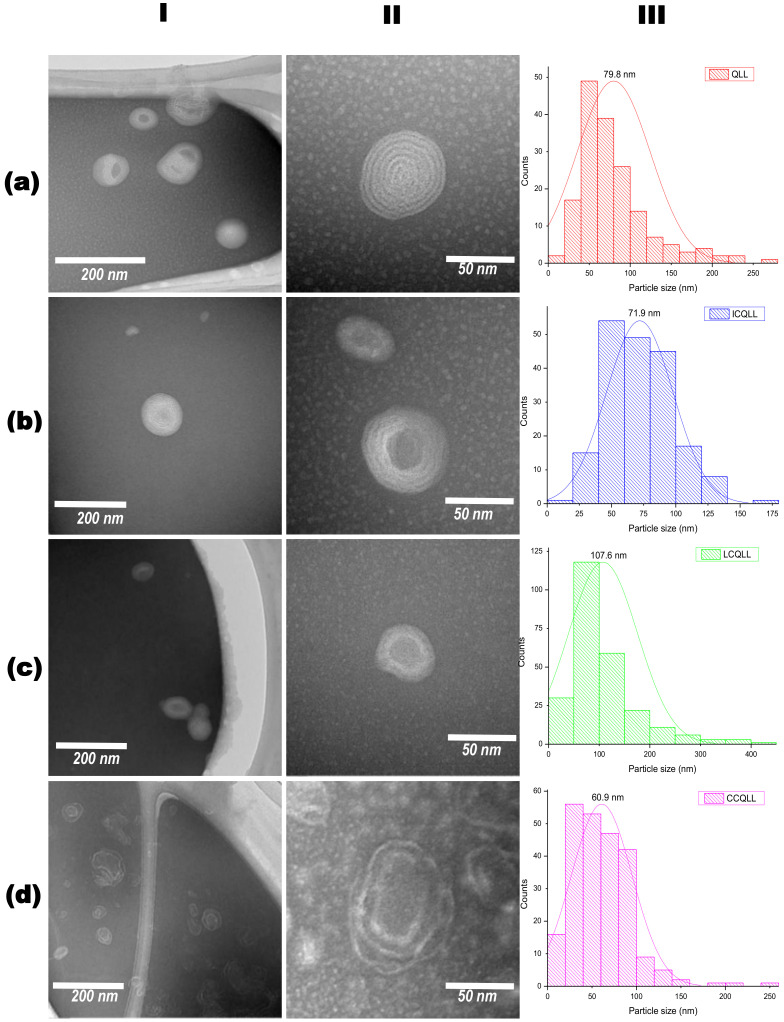
BF TEM images of uncoated and coated quercetin loaded liposomes. (**a**) QLL, (**b**) ICQLL, (**c**) LCQLL, (**d**) CCQLL. Column I and II show low and high magnification micrographs, respectively, column III shows the particle size distribution measured from the TEM micrographs.

Figure 3 shows bright field (BF) images acquired in the TEM of uncoated and coated quercetin- loaded liposomes. The first two columns show low and high magnification images, while the third column presents the particle size distribution determined from measuring at least 100 particles in the images. In general, the particles presented a distorted spherical morphology with sizes in the range of 10–500 nm. The average particle size was in the order of nanoparticles (below 100 nm), with values of 79.8, 107.6, 71.9, and 61.9 nm, for samples QLL, LCQLL, ICQLL, and CCQLL, respectively. With the exception of inulin, the other coating materials (lactose and chitosan) did not increase the primary particle size. High magnification images revealed the multilayer onion-like microstructure of the liposomes. This microstructure is well defined in the QLL showing about seven layers. With the addition of the polysaccharides, i.e., samples ICQLL and LCQLL, the multilayer microstructure begins to fade, observing the formation of a nucleus and the reduction in the number of layers (about 2–5 layers). The layered structure in the CCQLL is observed different to the onion microstructure of the other samples. In this sample, the distance between the layers increased and the layers are distorted, observed as incomplete spiral-like liposomes or a jellyfish-like microstructure. Hasan et al. described the microstructure of liposomes as alternate concentric dark and bright fringes, and suggested that quercetin is preferentially located in the bright fringes between two phospholipid layers in dark fringes [29]. In this sense, others works have reported that in the assembly of structures containing carotenoids, curcumin and quercetin, these biomolecules are placed between the phospholipid layers in the form of layered liposomes [29,45,46]. Nonetheless, Hao et al., proposed that quercetin was located in the core of liposomes rather than in the layers [23]. In the present work, a negative staining reagent was used to react preferentially with the unsaturated carbon-carbon bonds. From the molecules tested, i.e., quercetin, lecithin, inulin, lactose and chitosan, the first two presented unsaturation of carbon-carbon bonds, nevertheless quercetin has seven double bonds per molecule, while lecithin has only one. Therefore, in the unsaturated carbon-carbon bonds of quercetin the dyeing agent preferentially adsorbs, increasing the electronic scattering due to the high atomic number of molybdenum attached to antioxidant molecule, which is observed in the BF TEM image as an increase in contrast. Thus, the results presented herein suggested that quercetin is preferentially located between the two layers of lecithin. Additionally, the chemical nature of the molecules also supports the evidence aforementioned. For example, since quercetin is hydrophilic and lecithin is hydrophobic, the insoluble nature on aqueous media of quercetin causes to be located between the multilayer spaces of two phospholipid regions.

Regarding the observations done on the microstructure of CCQLL, it is possible that the acidic media used to maintain chitosan in soluble form, leads to a rearrangement of the phospholipid lamellar structure. This effect may be attributed to the high viscosity and stronger molecular interaction of this polymer with the liposome surface. Zhou et al. prepared liposomes loaded with vitamin C, obtaining irregularly shaped mono- and bilayer structured liposomes, and also noticed the increase in particle agglomeration after coating liposomes with pectin [47]. In consequence, polysaccharide-coated samples showed a more regular microstructure because lactose and inulin present neutral charges that lead to weaker attractions with the liposome walls [44].

### 3.4. Radical Scavenging Activity

**Figure 4:**
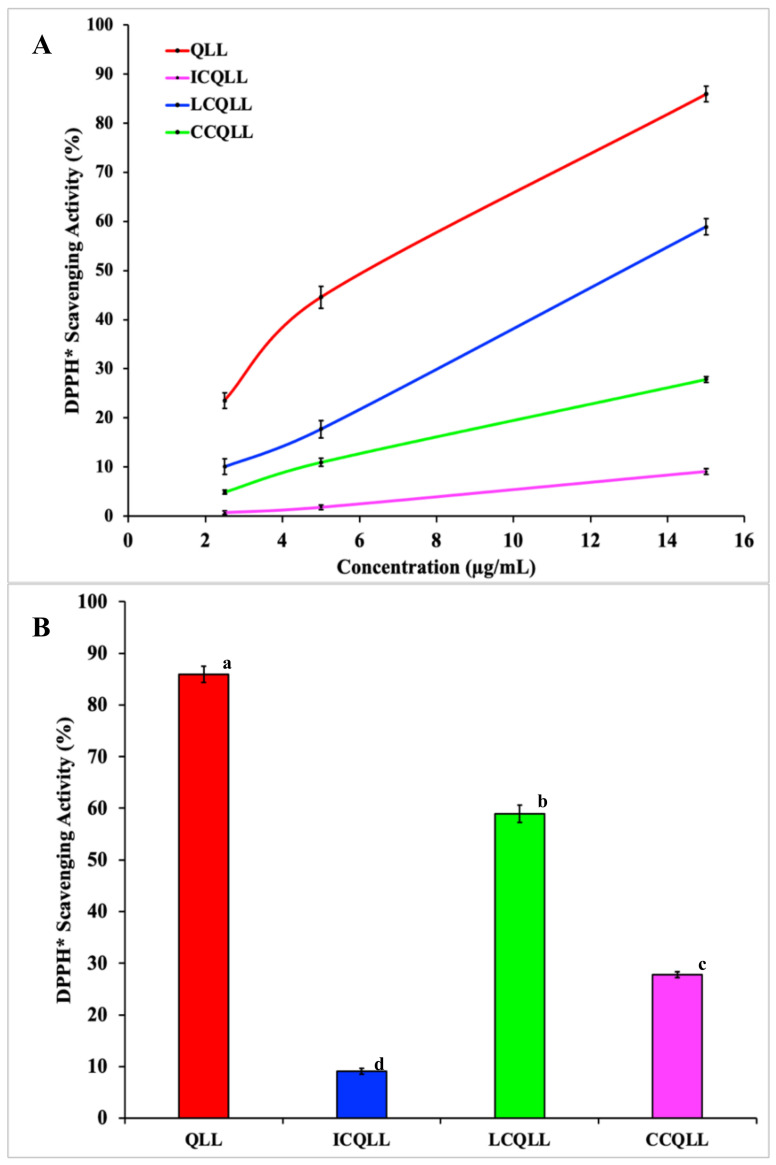
Effect of the coating material on the antioxidant activity of polysaccharide coated quercetin loaded liposomes. (**A**) Scavenging activity as a function of liposomes concentration. (**B**) Comparative scavenging activity at liposomes concentration of 15 µg/mL (a, b, c and d indicate the Tukey´s HSD test parameters).

Figure 4A,B shows the results of the DPPH radical scavenging activity (%) of uncoated and coated quercetin-loaded liposomes. Table 2 summarizes the average values of these experiments. Among the tested quercetin-loaded liposomes, the QLL sample showed the highest scavenging activity value, followed by LCQLL, and CCQLL, while the lower value was observed in the ICQLL sample. As expected, unloaded liposomes did not show any antioxidant activity, and for this reason were excluded from Figure 4A,B. The relatively high value presented by the QLL sample may result from the availability of quercetin in the particle, where the antioxidant is rapidly released upon dissolving in the aqueous ethanolic medium, promoting an immediate reaction between quercetin and the DPPH* radical. This was expected because of the ease of dissolution of liposomes in the alcoholic solution [48]. On the other hand, although the coating with polysaccharides may increase the stability of the liposomes, the coating material holds the quercetin inside the liposome structure, thus decreasing the antioxidant activity [49,50]. The susceptibility of liposomes to pH, enzymatic degradation, and loss of bio-activity during storage are some of the reasons for coating liposomes [45,51]. LCQLL showed lower antioxidant activity (27.5% less) than the QLL. This may result from the protective coating effect of lactose on the liposomal structure. Although lactose may prevent the easy dissolution of the lamellar liposomes structure, is partially permeable to release part of the quercetin in the alcoholic media. Additionally, because lactose is a molecule present on the surface of blood cells, its affinity avoids that macrophages capture the nanocarrier particles before they may achieve the target [34]. The Tukey’s HSD test parameters of the scavenging activity at 15 µg/mL (Figure 4B) showed statistically significant (p = 0.05) differences of CCQLL and ICQLL samples in comparison to LCQLL, indicating those samples presented lower antioxidant activity.

However, there is not a clear trend in the different results reported in the literature. Zhao et al. prepared chitosan-loaded liposomes with coenzyme Q10 and α-lipoic acid. They found a hydroxyl radical scavenging of 59% for the uncoated liposomes, and 64% for chitosan-coated liposomes, and explained that this increase may be attributed to the protecting effect of amino groups of chitosan against hydroxyl radical [52]. Caddeo et al., prepared quercetin-loaded liposomes coated with Eudragit^®^ S100. The antioxidant activity for quercetin solution was 90%, 81% for quercetin-loaded liposomes, and 78% for Eudragit^®^ S100 coated quercetin-loaded liposomes [53]. Hao et al. found slight differences in the antioxidant activity for quercetin-loaded liposomes coated and uncoated with chitosan [23]. Thus, it is evident that other factors such as microstructure, molecular weight distribution, and stearic hindrance of the coating material influence the chemical interactions within the antioxidant molecule, thus affecting the antioxidant activity. From the microstructural results observed by TEM, the CCQLL particles showed a distorted microstructure with a spiral shape, while in the ICQLL the particles were observed to be more ordered. These differences in the microstructure may help to explain the antioxidant activity. In the CCQLL, the release of quercetin is less restricted by the disordered microstructure, which acts as a weak barrier. While in the ICQLL, in addition to the microstructural order, the higher molecular weight may also reduce the release of quercetin. These features may act as a strong barrier avoiding that the radicals interact with the antioxidant. This observation also supports the results found in the LCQLL sample. Although the microstructural order in lactose-coated samples is greater than that of inulin, the molecular weight of lactose is much lower. This suggests that molecular weight plays an important role in the antioxidant release of coated quercetin loaded liposomes, where a higher molecular weight restricts the release of the antioxidant. Takeuchi et al. prepared coated liposomes with modified polyvinyl alcohol (PVA-R) with different molecular weights [54]. They found that the thickness of the liposomes was related with the molecular weight of the PVA-R, where the higher molecular weight resulted in thicker and more resistant liposomes, that last more time within the bloodstream of rats.

**Figure 5:**
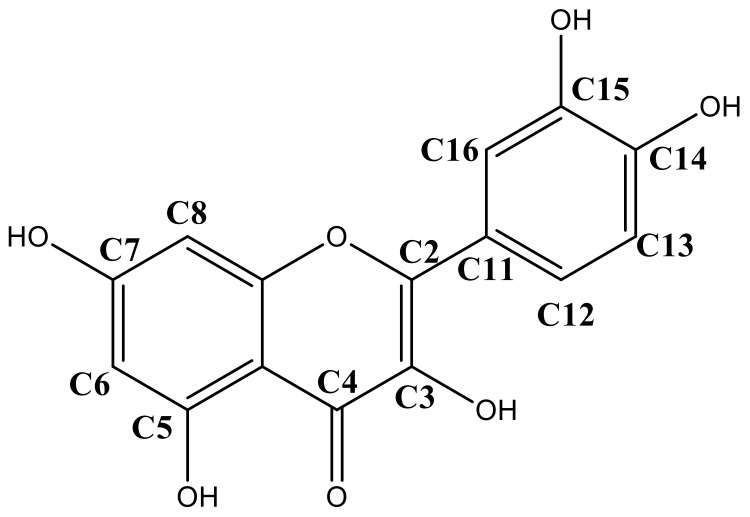
Quercetin molecule showing the relative positions of its hydroxyls.

In addition, the chemical structure of quercetin also plays a major role in the chemical interactions with the different polysaccharide. Figure 5 depicts the chemical structure of quercetin showing the relative position of hydroxyl groups (OH) were chemical interactions can occur. The hydroxyls from carbons C7, C14 and C15 are available to establish strong hydrogen bond-type interactions with the polar groups of polysaccharides. Hydroxyls from carbon C3 and C5 also may establish hydrogen bond interactions, but the close position relative to the carbonyl group at C4 limits their availability to interact with external polar groups [55]. From the three polysaccharides tested in the present work, lactose is a disaccharide, while chitosan and inulin are polysaccharides. Thus, the molecule size i.e., molecular weight distribution, and the number of hydroxyls available for chemical interactions, also influences the release of quercetin in the alcoholic solution. Leyva-Porras et al. showed that inulin with a higher degree of polymerization (DP) adsorbed more water than the low DP inulin in the whole range of water activities (a_w_) [56]. Araujo-Díaz et al. reported a better performance of maltodextrin in the conservation of resveratrol and quercetin when compared with inulin [36]. Besides the differences in chemical structure between these two polysaccharides, the DP was 2-12 for inulin and 2-16 for maltodextrin. In this sense, low DP maltodextrins have shown a better performance in the content of quercetin than high DP maltodextrins [57].

Recently, Leyva-Porras et al., qualitatively compared the interaction of maltodextrins with quercetin and resveratrol, and concluded that the number of active sites for chemical interactions is more important than chemical hindrance in these molecules [58]. From the above it is possible to infer that polysaccharides with relatively high molecular weight distribution such as chitosan and inulin tend to trap the quercetin within the liposome, while in lactose-coated liposomes, and due to the low molecular weight of the disaccharide, the antioxidant is more available for interactions with the DCCP molecule, hence the higher antioxidant activity.

Agglomeration occurs when the material is dried i.e. for morphological studies. While the material was in solution, agglomeration was not noticed, indicating the suspension of liposomes in solution. According to Hao et al. [23], particle growth was observed (although it was not identified as agglomeration) and apparently did not affect the performance in the antioxidant properties. In the work reported by Li et al. [42] agglomeration was observed, but the agglomerated materials were discarded prior the drug release tests. In the present work, there was not a noticeable trend between the agglomerated particles and the performance as antioxidant. The liposomes coated with chitosan presented the largest particle size (identified as agglomeration) and an intermediate antioxidant behavior. Conversely, the antioxidant behavior of lactose and inulin coated liposomes, which showed relatively smaller particle size (i.e., less agglomerated than those coated with chitosan), presented the higher and lower antioxidant activities, respectively. Then, the differences in the antioxidant performance may be attributed to the chemical interactions of quercetin, and the microstructural disorder induced by the coating polysaccharides, rather than to the agglomeration of liposomes in solution.

## 4. Conclusions

Three polysaccharides (lactose, chitosan and inulin) were employed as coating materials in the preparation of quercetin-loaded liposomes (QLLs). Coated and uncoated liposomes were characterized and tested in the scavenging of free radicals in solution. Particle size determined by light scattering showed large particle sizes in the range of 200 nm for QLL, LCQLL, and ICQLL, while it was 600 nm for CCQLL. This was corroborated by scanning electron microscopy (SEM) as due to the agglomeration of pseudo-spherical particles. However, from transmission electron microscopy (TEM) the microstructure revealed the primary particle size of the coated liposomes below 100 nm, and the layered structure formed of at least two layers. The antioxidant activity of QLL showed a better performance that the coated liposomes. This was explained in terms of quercetin availability since in the coated liposomes, the polysaccharides trap the quercetin between the layers, reducing its availability for radical scavenging. Among the three coating materials, lactose presented the higher antioxidant activity. This may be caused by the relatively smaller size of the disaccharide when compared with the other two polysaccharides (inulin and chitosan) which are considered as carbohydrate polymers. This suggested that the higher the molecular weight of the polysaccharide, the higher the chemical interactions with the quercetin inside the liposome, observed as the retention of the antioxidant and consequently a decrease in the antioxidant activity. This study opens the possibility of using polysaccharides with different potential properties for coating liposomes in the conservation and release of antioxidants.

## Figures and Tables

**Table 1:** Samples identification and description of liposomes. (CL) control liposomes, (QLL) quercetin loaded liposomes, (XCCL) polysaccharide coated control liposomes, and (XCQLL) polysaccharide coated quercetin loaded liposomes. Where X stands for the corresponding polysaccharide (I) inulin, (L) lactose, and (C) chitosan.

Sample Name	Coating	Quercetin Loaded
CL	Uncoated	No
ICCL	Inulin	No
LCCL	Lactose	No
CCCL	Chitosan	No
QLL	Uncoated	Yes
ICQLL	Inulin	Yes
LCQLL	Lactose	Yes
CCQLL	Chitosan	Yes

**Table 2:** Summary of antioxidant activity of uncoated and polysaccharide coated quercetin loaded liposomes at different concentrations.

Sample	Scavenging Activity (%)		
	15 µg/mL	5 µg/mL	2.5 µg/mL
QLL	85.95	44.54	23.53
ICQLL	9.06	1.78	0.71
LCQLL	58.90	17.73	10.08
CCQLL	27.83	10.93	4.85
CL	----	----	----
ICCL	----	----	----
LCCL	----	----	----
CCCL	----	----	----
